# Mitochondria-associated endoplasmic reticulum membranes and cardiac hypertrophy: Molecular mechanisms and therapeutic targets

**DOI:** 10.3389/fcvm.2022.1015722

**Published:** 2022-10-20

**Authors:** Yi Luan, Yage Jin, Pengjie Zhang, Hongqiang Li, Yang Yang

**Affiliations:** ^1^Clinical Systems Biology Research Laboratories, Translational Medicine Center, The First Affiliated Hospital of Zhengzhou University, Zhengzhou, China; ^2^Department of Cardiology, The First Affiliated Hospital of Zhengzhou University, Zhengzhou, China; ^3^Department of Ultrasound, The First Affiliated Hospital of Zhengzhou University, Zhengzhou, China; ^4^Department of Thyroid Surgery, The First Affiliated Hospital of Zhengzhou University, Zhengzhou, China

**Keywords:** cardiac hypertrophy, mitochondria-associated ER membranes (MAMs), MAMs-associated proteins, cardiovascular system, therapeutic targets

## Abstract

Cardiac hypertrophy has been shown to compensate for cardiac performance and improve ventricular wall tension as well as oxygen consumption. This compensatory response results in several heart diseases, which include ischemia disease, hypertension, heart failure, and valvular disease. Although the pathogenesis of cardiac hypertrophy remains complicated, previous data show that dysfunction of the mitochondria and endoplasmic reticulum (ER) mediates the progression of cardiac hypertrophy. The interaction between the mitochondria and ER is mediated by mitochondria-associated ER membranes (MAMs), which play an important role in the pathology of cardiac hypertrophy. The function of MAMs has mainly been associated with calcium transfer, lipid synthesis, autophagy, and reactive oxygen species (ROS). In this review, we discuss key MAMs-associated proteins and their functions in cardiovascular system and define their roles in the progression of cardiac hypertrophy. In addition, we demonstrate that MAMs is a potential therapeutic target in the treatment of cardiac hypertrophy.

## Introduction

As an adaptive response to hemodynamic stress, cardiac hypertrophy compensates cardiac performance, and improves ventricular wall tension and oxygen consumption. The hypertrophic alterations in the heart are accompanied by several heart diseases, which include ischemia disease, hypertension, heart failure (HF), and valvular disease. In these cardiac pathologies, cardiac hypertrophy induced by pressure overload confers compensatory effect by ameliorating wall stress and oxygen consumption. Meanwhile, ventricular hypertrophy has been shown to increase the risk of HF and malignant arrhythmia.

Cardiac hypertrophic transformation is divided into three stages: (1) hypertrophy developing, which means load exceeds output; (2) hypertrophy compensation, which refers to the stage where the exceeded workload/mass ratio is normalized and cardiac output is maintained during resting; and (3) overt HF, which indicates ventricular dilation and progressive reduction in cardiac output despite persistent activation of hypertrophic program. Late HF is accompanied with perturbated calcium homeostasis and ionic currents, which lead to poor prognosis of ventricular dysfunction and malignant arrhythmia. Cardiac hypertrophy is characterized by enhanced cell size and protein synthesis, and elevated organization of sarcomere. Cardiac hypertrophy phenotypes can be categorized into concentric hypertrophy and eccentric hypertrophy. Concentric hypertrophy is induced by pressure overload, featured by parallel organization of sarcomeres and lateral formation of cardiomyocytes, while eccentric hypertrophy is caused by volume overload or prior infarction and is characterized by continuous addition of sarcomere and longitudinal cell growth. These cellular alterations are associated with decline of the fetal gene program, whose expression mimic those of embryonic development.

The pathogenesis of cardiac hypertrophy is complicated and its progression evolves multiple cellular processes. Besides, the mechanisms of cardiac hypertrophy remain elusive. Mitochondria dysfunction has been implicated in the development and pathogenesis of cardiac hypertrophy. In addition, previous studies have shown that these hypertrophic processes influence many mitochondrial processes. Apart from the mitochondria, endoplasmic reticulum (ER) dysfunction may also be involved in the development of cardiac hypertrophy. Previous evidence has shown close interaction between the mitochondria and ER, which is mediated by mitochondria-associated ER membranes (MAMs), or mitochondria-ER contact sites (MERCs) (1, 2). In addition, recent studies have demonstrated the function of the MAMs in the regulation of cardiovascular diseases and could act as potential therapeutic targets in cardiovascular diseases (3). In this review, we discussed the components in MAMs and MAMs-associated proteins and define their roles in cardiac hypertrophy. We also demonstrate the potential application of MAMs as a therapeutic target in cardiac hypertrophy.

## Composition of the mitochondria-associated membranes

MAMs were first described in the late 1950s, which were isolated and examined in 1990s (4, 5). Previous studies have performed proteolytic and proteome analyses of the MAMs (6, 7). To date, approximately 1,000 proteins localized within MAMs have been identified in the brain and liver using in-depth mass spectrometry analysis (8). MAMs components are highly conserved in many species and tissues (9). The functions of the MAMs were also explored and showed to be associated with calcium transfer, lipid synthesis, autophagy and reactive oxygen species (ROS) (10). Besides, MAMs are also involved in inflammasome formation, ER stress and mitochondria dynamics (11).

MAMs-localized proteins either mediate the physical interactions of MAMs, or regulate tethering complexes in MAMs (12). Tethering proteins range from Ca^2+^ channels to apoptotic proteins and act as molecular bridges between the ER and the mitochondrial membrane (13). The length of the MAMs varies from 10 to 25 nm for smooth ER, and 50 to 80 nm for rough ER. As for the coverage, MAMs occupy approximately 4 to 20% of the total mitochondrial surface, depending on the metabolic condition and cellular stress (7).

**FIGURE 1:**
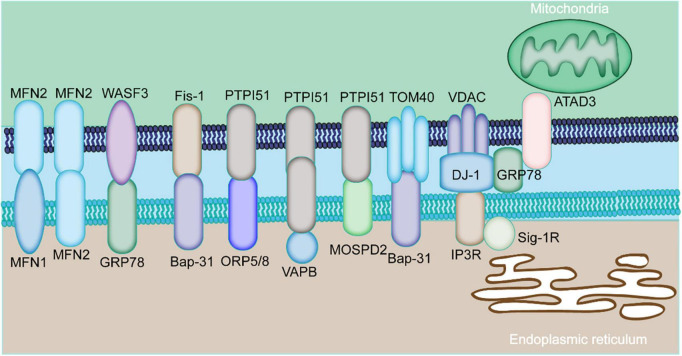
Major components of MAMs proteins. The physical interaction among MAMs is mediated by various proteins expressed by MAMs. The mitochondria-localized MFN2 forms a hetero- or homo-dimer with MFN1 and MFN2 on the ER. The ER chaperone GRP78 interacts with WASF3. The ER-localized BAP31 interacts with the mitochondrial Fis1 and TOM40. The mitochondrial PTPIP51 interacts with the ER protein ORP5/8, VAPB, or MOSPD2. ER-resident IP3R is anchored to OMM-localized protein VDAC *via* GRP75. The ER protein, GRP78, cytoplasm-residing WASF3, the IMM localizing ATAD3A constitute the GRP78-WASF-ATAD3A complex.

The MAMs proteins can be classified into pro-tethering complexes, proteins that modulate IP3Rs/GRP75/VDAC complexes, anti-tethering factors and upstream regulators of MAMs assembly. The pro-tethering complexes include; (i) mitofusin 2 (MFN2), (ii) vesicle-associated membrane protein-associated protein B (VAPB) and protein tyrosine phosphatase interacting protein 51 (PTPIP51) complex, (iii) PTPIP51 and motile sperm domain-containing protein 2 (MOSPD2), (iv) glucose-regulated protein 75 (GRP75) bridging inositol triphosphate receptor (IP3R) to voltage-dependent anion channel 1 (VDAC1) complex, (v) the mitochondrial fission 1 protein (Fis1-) B cell receptor-associated protein 31 (BAP31) complex, (vi) the FUN14 domain containing 1 (FUNDC1-) IP3R2 complex, (vii) the phosphofurin acidic cluster sorting protein 2 (PACS2), (viii) PDZD8, (ix) Beclin1 (BECN1), and (x) mitochondrial ubiquitin ligase (MITOL), Parkin, and AMPKα, which modulate formation of MAMs through direct interaction with MFN2 on the outer mitochondrial membrane (OMM). Those proteins that modulate IP3Rs/GRP75/VDAC complexes include (i) sigma-1 receptor (Sig-1R), mitochondrial translocase of the outer membrane 70 (TOM70), (ii) cyclophilin D (CypD), (iii) pyruvate dehydrogenase kinases 4 (PDK4), (iii) thymocyte-expressed, positive selection-associated gene 1 (Tespa1), (iv) reticulon 1C (RTN-1C), (v) glycogen synthase kinase-3β (GSK3β), (vi) disrupted-in-schizophrenia 1 (DISC1), (vii) transglutaminase type 2 (TGM2), (viii) wolfram syndrome 1 (WFS1), and (ix) etoposide-induced protein 2.4 (EI24). On the other hand, anti-tethering factors include trichoplein/mitostatin (TpMs) which suppresses MAMs tethering *via* MFN2, (ii) FATE1 dissociates from MAMs *via* interaction with ER chaperones and emerin (EMD) and the mitofilin, as well as (iii) Caveolin-1. Upstream regulators of MAMs assembly include (i) GSK3β, (ii) p38 MAPK, (iii) cGMP-dependent protein kinase (PKG), (iv) FOXO1, (v) cAMP-dependent protein kinase (PKA), and (vi) AMPKα (Figure 1) (14).

The MAMs proteins are grouped into three categories based on their localization: (1) proteins that are only localized on the MAMs; (2) proteins expressed on the MAMs and other cellular compartment; (3) proteins temporally enriched on the MAMs under certain circumstances (15). The specific characterization of MAMs-related proteins is currently unknown given their high dynamic nature.

The IP3R1-GRP75-VDAC1 complex is the first tethering complex to be discovered and is composed of the ER-localized IP3R1s and OMM-localized VDAC1. This complex regulates calcium release from the ER to the mitochondria (16). The GRP75 has been shown to be physically associated with IP3R1 and VDAC1 (16). In cardiomyocytes, OMM-residing FUNDC1 was found to directly bind to IP3R2, forming a bridge between ER and mitochondria, which promotes the transfer of calcium to the mitochondria (17). Depletion of FUNDC1 induced ubiquitination and clearance of IP3R2 and reduced PACS2 expression level, a key regulator of MAMs (18). Abnormal expression level of IP3R has been implicated in multiple cardiac disorders, like cardiac hypertrophy, failing myocardium, atrial fibrillation (Table 1) (19). Several other proteins have been found to regulate IP3R-VDAC. Sig-1R interacts with ER chaperons, like BiP and prolongs Ca^2+^ release from the ER into mitochondria by stabilizing IP3R3 at MAMs (20). PDK4 interacts with the GRP75-IP3R-VDAC complex and stabilizes this complex at MAMs. Inhibition of PDK1 reduced the formation of MAMs, prevented calcium accumulation in the mitochondria of skeletal muscle (21).

**TABLE 1:** Components of MAMs involved in cardiac diseases.

Proteins	Relevant function(s) in MAMs	Functions in cardiac diseases
**Protethering proteins**		
GRP75	Increased MAMs formation and mitochondria Ca^2+^ uptake	Mitochondrial calcium overload and hypoxia/reoxygenation injury in cardiomyocytes.
IP3Rs	Interact with GRP75 and VDACs, modulate calcium in MAMs	Upregulation in cardiac hypertrophy. Modulate excitation-contraction coupling in ventricular and atrial cardiomyocytes.
VDACs	Interact with GRP75 and IP3Rs, regulate intracellular Ca^2+^ level	Marked elevation of VDAC1 in myocardial infarction.
MFN2	Modulator of ER-mitochondria tethering and mitochondrial fusion	Downregulation in cardiac hypertrophy. MFN2 upregulation ameliorated the cardiac hypertrophy.
MFN1	Tethering mitochondria to MAMs *via* interaction with ER-resident MFN2	Repress cardiac hypertrophy.
Fis1	Modulate ER-mitochondria tethering and induce apoptosis. Induce mitophagy	Inhibition of the CREB/Fis1 pathway leads to heart disease.
BECN1	Enhance MAMs formation and autophagosomes	Deregulation leads to heart diseases.
FUNDC1	Promote mitochondrial fission and mitophagy. Increase Ca^2+^	Required for cardiac ischemia reperfusion injury.
Parkin	Mediate mitophagy. Increase the ER-mitochondria contacts, induce Ca^2+^ transfer and ATP synthesis.	Upregulated during I/R injury.
**IP3Rs/GRP75/VDAC complex-modulated proteins**		
Sig-1R	Prolong Ca^2+^ signaling; Sig-1R increase represses ER stress response, whereas Sig-1R decrease induces apoptosis	Sig-1R activation represses hypertrophy. Sig-1R KO displays cardiac remodeling.
CypD	Regulates Ca^2+^ transfer from the ER to mitochondria through IP3R1	The CypD/GRP75/IP3R/VDAC complex inhibition improved hypoxia/reoxygenation injury.
GSK3β	Inhibition of GSK3β results in decreased ER Ca^2+^ release as well as sensitivity to apoptosis	GSK-3β inhibition reduced infarct size in reperfused hearts.
**Antitethering proteins**		
CAV1	Negatively regulate the formation of MAMs and impair Ca^2+^ transfer	CAV1 ablation decreases survival in myocardial ischemia.
**Upstream regulators of the formation of MAMs**		
p38 MAPK	p38 MAPK inhibits MAMs formation and mitochondrial fusion by promoting degradation of MFN1/2	p38 MAPK has been implicated in cardiomyocyte dysfunction and apoptosis
FOXO1	Augment MAMs formation and promote mitochondrial Ca^2+^ accumulation, mitochondrial dysfunction, and ER stress	FOXO1 protein is associated with ischemic heart disease.

Mitofusin 2 is well known for its role in mitochondria fusion. It forms homo- or hetro-dimers with MFN1 in the OMM (22, 23). MFN2 knockdown enhanced the contact between ER and mitochondria and increased calcium transfer, implying it is not a physical tether (24). However, MFN2 inhibition also amplifies the distance between ER and mitochondria and reduces the transfer of calcium to the mitochondria (25). Generally, MFN2 is considered to be a key component that determines the normal function of MAMs. For instance, MFN2 expression was found to be reduced in rat models of cardiac hypertrophy, and rats with spontaneous hypertension, transverse aortic banding, and myocardial infarction. Overexpression of MFN2 suppressed cardiac hypertrophy caused by angiotensin II (26). In addition, MFN2 regulates cardiac development and differentiation in embryonic stem cells (27).

The BAP31-Fis1 complex is composed of ER-residing BAP31 and OMM-residing Fis1, which facilitates recruitment and activation of procaspase 8, bridging two critical organelles for apoptosis signaling (28). During apoptosis, caspases and their cleavage products cleave BAP31 to form p20BAP31 which then transmits the apoptotic signal *via* the IP3 receptor complex to the ER-mitochondria contact (29). Moreover, BAP31 regulates mitochondrial oxygen consumption, autophagy, and mitochondrial homeostasis through TOM40, mitochondrial respiration complex, and NADH ubiquinone oxidoreductase (mitochondrial complex 1) core subunit 4 (NDUFS4) (29). Therefore, BAP31 is an essential factor in the transmission of apoptotic signal between the ER and mitochondria. The synaptojanin-2 binding protein (SYNJ2BP) and ribosome-binding protein 1 (RRBP1) complex have been found to regulate mitochondria-ER interactions. Overexpression of SYNJ2BP enhanced the contact between mitochondria and ER (30).

The VAPB-PTPIP51 complex consists of VAPB localized on the ER and PTPIP51 localized on the OMM. VAPB modulates vesicle trafficking and unfolded protein response, a reaction of the ER to suppress accumulation of misfolded proteins, whereas PTPIP51 modulates cellular development and tumorigenesis (31). Abnormal interaction between VAPB and PTPIP51 may decrease MAMs expression and interfere with calcium handling to delay mitochondrial calcium uptake (32). Aberrant expression of PTPIP51 or VAPB has been associated with alterations in MAMs distribution on the mitochondria surface (32). Several other proteins have been shown to modulate the interaction between VAPB and PTPIP51 complex. For instance, the α*-Synuclein* mutant can disrupt the interaction of VAPB and PIPIP51 leading to a reduction in the mitochondria-ER contact, diminished calcium transfer, and decreased mitochondrial ATP production during the pathogenesis of Parkinson’s disease (31). Another highly conserved nuclear protein, TAR DNA-binding domain protein 43 (TDP-43) was reported to modulate VAPB-PTPIP51 interaction (33). The accumulation of TDP-43 abrogates the VAPB-PTPIP51 interaction and calcium homeostasis by promoting the phosphorylation and activation of glycogen synthase kinase-3β (GSK-3β) (33, 34). In addition, PTPIP51 interacts with oxysterol-binding protein-related protein 5/8 (ORP5/8) at the ER membrane to enhance the transfer of the phosphatidylserine (PS) to the mitochondria (32). Elsewhere, it was reported that PTPIP51 interacted with the ER-anchored MOSPD2 to influence intracellular exchange and communication (35).

The ER protein, GRP78, cytoplasm-residing WASF3, the inner mitochondrial membrane (IMM) localized ATPase family AAA domain-containing 3A (ATAD3A) form the GRP78-WASF-ATAD3A complex by penetrating the OMM and binding to ATAD3A (36). Studies have shown that ATAD3A contributes to the invasion of breast and colon cancer cells *via* regulating GPR78-mediated stabilization of WASF3 (36, 37). Moreover, ATAD3A tethers to other OMM and ER localizing proteins, such as MFN2, dynamin-related protein 1 (Drp1), and BiP *via* the cytosolic protein WASF3 (38). Apart from the aforementioned protein complexes, many other MAMs-related regulatory proteins have been reported. The interaction between TG2 and GRP75 regulates the ER-mitochondrial Ca^2+^ flux and profile of the MAMs proteome (39). There is a need to comprehensively study the proteins associated with the formation of the mitochondria-ER contact and identify other tethers and spacers in this process.

## The cellular processes mediated by MAMs in cardiac hypertrophy

MAMs are involved in multiple cellular processes, including calcium homeostasis, lipid exchange, mitochondrial physiology, mitophagy, ER stress, and inflammation (40). The detailed roles of these MAMs are discussed in the following chapters.

### Calcium transfer

**FIGURE 2:**
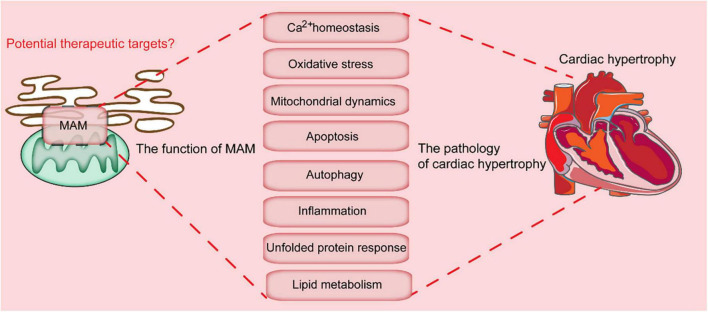
Key cellular processes mediated by MAMs-localized proteins. MAMs are involved in multiple cellular processes, which include calcium homeostasis, oxidative stress, mitochondrial dynamics, apoptosis, autophagy, inflammation, unfolded protein response, and lipid metabolism. Dysregulation of these processes leads to the progression of cardiac hypertrophy.

Calcium transfer between the ER and the mitochondria affects both the heart and vascular system (11, 41). Calcium transfer from the ER to the mitochondria modulates apoptotic processes and energy production *via* the MAMs. Of note, IP3R3-GRP75-VDAC1 remains the main tethering complex that mediates calcium release from the ER to the mitochondria (16). IP3R1 accumulates high concentration of calcium in nearby of ER. VDAC1 acts as a calcium channel in the OMM while GRP75 acts as a bridging compartment to tether into the VDAC1/GRP75/IP3R1 channel complex (11). This complex also functions as a molecular scaffold for other calcium modulators, such as Sig-1R, BiP, Bcl-2, and IRBIT (Figure 2) (42, 43). Previous data has demonstrated abnormal expression of IP3Rs in cardiac hypertrophy, implying the inductive role of IP3Rs in cardiac hypertrophy (17, 18). Moreover, IP3Rs also mediate excitation-contraction coupling both in ventricular and atrial cardiomyocytes (19). In addition, Sig-1R, a calcium modulator, modulates calcium transfer between the mitochondria and the ER by maintaining the stability of the IP3R (20). FUNDC1 can regulate ER Ca^2+^ transfer into mitochondria by interaction with IP3R2, and lead to aberrant mitochondrial fission, and finally to cardiac dysfunction and HF.

RNA-dependent protein kinase (PKR)-like ER kinase (PERK), a key ER stress protein, is also involved in calcium transfer, maintenance of ER morphology, and MAMs formation (44). A lack of the PERK protein leads to reduced mitochondrial calcium uptake and impaired calcium homeostasis, which is positively correlated to mitochondrial ATP production (45, 46). In the contrary, excessive calcium uptake in the mitochondria leads to calcium overload and oxidative stress (47). Therefore, a precise mechanism of MAMs-mediated calcium transfer between the ER and the mitochondria is essential for the normal functions within the cells.

### Lipid synthesis and exchange

Lipid synthesis is an essential cellular process which maintains the cell membrane, cell signaling transduction, and synaptic transmission (48, 49). Although lipid synthesis mainly occurs in the ER, other organelles, such as mitochondria also play indispensable roles (50). Many enzymes responsible for lipid synthesis are localized on the membrane of the mitochondria. MAMs enriched proteins involved in phospholipid synthesis and transport include diacylglycerol O-acyltransferase 2 (DGAT2), fatty acid CoA ligase 4 (FACL4), phosphatidylethanolamine N-methyltransferase 2 (PEMT2), cholesterol acyltransferase/sterol O-acyltransferase 1 (ACAT1/SOAT1), as well as PSS1 and PSS2 (Figure 2) (49, 51). FACL4 is one of the most reliable MAMs markers and is involved in the synthesis of triacylglycerols (52). ACAT1 is involved in the formation of cholesteryl esters from free cholesterol, and sustains dynamic homeostasis of membrane-localized and cytoplasmic lipid droplets stored cholesterol (53). MAMs-associated caveolin-1 (CAV1) modulates cholesterol efflux by binding to VDAC2. Suppression of the CAV1 is associated with aberrant intracellular accumulation of free cholesterol, reduced physical extension and integrity of the MAMs (54).

In addition, MAMs-localizing proteins mediate formation of biological membranes, phosphatidylcholine, phosphatidylethanolamine (PE), and phosphatidylserine. Phosphatidylserine synthesis is mediated by PSS1 and PSS2 on the MAMs, and then transferred to the mitochondria by ORP5/8-PTPIP51 tethering complex (55), which is then catalyzed into PE in the IMM by a decarboxylase enzyme. The newly formed PE is then converted into phosphatidylcholine (PC), a major component of the cell membrane, by MAMs-enriched PEMT2 in the mitochondria. The phospholipid acids are generated in the ER and transferred to the mitochondria for modification of cardioprotective cardiolipin, which is beneficial for the stability and activity of the mitochondrial calcium uniporter (56). MAMs are also involved in the production of ceramide, which is a bioactive sphingolipid and important in modulating cell growth. The alterations in the MAMs-localizing proteins greatly influence lipid anabolism. The deficiency of ORP8 leads to altered HDL biosynthesis (57). In addition, *ATAD3* gene cluster ablation in human fibroblasts has been shown to interfere with cholesterol and lipid metabolism (39).

### MAMs modulate endoplasmic reticulum stress

When misfolded proteins aggregate in the ER lumen, it disrupts ER homeostasis which induces ER stress (58). The ER stress is initiated by ER stress sensor protein kinase PERK, ATF6, and IRE1α (59). These proteins are maintained in an inactive state by GPR78, which is induced in many cardiac disorders, such as dilated or ischemic cardiomyopathy. The ER stress signaling is regulated by MAMs tethering complex, while the PERK activity is affected by MFN2 (Figure 2) (60). PERK is associated with the intersection between the mitochondria and the ER, and ROS-induced mitochondrial apoptosis. Activation of PERK leads to subsequent activation of the PERK-EiF2α-ATF4-CHOP pathway (61). VAPB, another MAMs protein, interacts with ATF6 to directly inhibit UPR (59). The enrichment of IRE1α in MAMs either induces cell survival or cell death by fueling mitochondrial Ca^2+^ overload (59). Besides, IRE1α ubiquitylation at MAMs hinders ER stress-induced apoptosis (62). On the other hand, cardiac specific depletion of XBP1 induces significant increase in myocyte death and pathological remodeling in mice. In addition, depletion of other MAMs-enriched proteins such as PACS2, Sig-1R, MFN2, or CypD promotes ER stress by disrupting the ER-mitochondria contact.

### Regulation of oxidative stress

Reactive oxygen species is necessary for maintenance of cellular homeostasis (63). Excess production of ROS, especially in the mitochondria, leads to oxidative damage to proteins, lipids and DNA (64). Mitochondrial ROS (mtROS) can be induced by excessive calcium transfer by the MAMs. The formation of MAMs in podocytes can be induced by diabetes, which leads to excessive calcium transfer and mtROS abundance. Conversely, reduced MAMs formation by FUNDC1 depletion alleviates mtROS accumulation, thus confirming the correlation between the MAMs and mtROS (17). Moreover, MAMs-enriched calcium channels are responsible for modulation of calcium and mtROS production. Oxidoreductin-1 α (Ero1-α), and ER resident protein 44 (ERp44), which are highly enriched MAMs oxidoreductases in the ER, can induce excess production of mtROS (65). Ero1-α induces calcium transfer from the ER to the mitochondria and leads to excessive generation of mtROS by inducing IP3R1 oxidation and ERp44 from IP3R1 (66). DsbA-L, a multifunctional protein, localized in the mitochondrial matrix, ER and MAMs fraction, is also involved in the production of mtROS (67). The inductive modulation of p66Shc on mtROS production and mitochondrial fission is attributed to Ser36 phosphorylation. Besides, the phosphorylation is transferred to the MAMs, contributing to mtROS generation (68). In conclusion, these MAMs-enriched proteins play a key role in maintaining the mtROS homeostasis. However, data on the detailed roles of mtROS in the pathogenesis of cardiac diseases, such as cardiac hypertrophy remain scant and thus require further analysis.

### Mitochondrial physiology

Apart from the above-mentioned cellular processes, MAMs are also implicated in mitochondrial physiology, including mitochondrial bioenergetics, dynamics, and mitophagy (Figure 2). Since the heart is an organ that is highly dependent on energy, there is high concentration of mitochondria in the heart tissues. Abnormal mitochondria function contributes to various cardiac diseases (69). On the other hand, mitochondrial dynamics include mitochondrial fission, fusion, and motility.

#### Mitochondrial fission

During mitochondrial fission, there is formation of a helix adjacent to the mitochondria, which constricts and divides mitochondria into two parts, following Drp1 recruitment onto the OMM. The recruitment of Drp1 requires the assistance of its receptors, Fis1, mitochondrial fission factor (Mff), and mitochondrial dynamics proteins (MiD49 and MiD51), which localizes at the ER-mitochondria interface (70). Binding of the Drp1 to the F-actin activates the Drp1 GTPase activity which leads to recruitment of the Drp1 into the pre-constricted mitochondria to stimulate its fission (71). FUNDC1, a new Drp1 receptor, can facilitate mitochondria fission by binding to calnexin, an ER-localizing protein, in response to hypoxia (18). Drp1 mutation in mice leads to cardiomyopathy accompanied with spotty calcifications in heart tissues (72). The inhibitor of mitochondrial division/mitophagy (Mdivi) ameliorates pressure-overload induced HF. Other MAMs-residing proteins, such as inverted formin 2 (INF2), syntaxin 17 (STX17), and Rab32 are involved in the regulation of mitochondria fission. Besides, INF2 mediates mitochondrial constriction and division by interacting with actin-nucleating protein Spire, Spire 1C (73).

#### Mitochondrial fusion

In general, mitochondrial fusion is induced by the OMM proteins, MFN and the IMM protein optic atrophy 1 (OPA1). MFN2 mediates mitochondrial tethering and fusion, and its deficiency protects from ischemia/reperfusion damage in mice (74, 75). In addition, tethering of MFN2 with the MITOL, maintains mitochondrial dynamics (Figure 2) (76). Besides, mitochondrial fusion facilitates material exchange between impaired and healthy mitochondria, which restores the normal functioning of the mitochondria. A recent study proposed that fusion and fission begin at the contact site between ER and mitochondria which sustains the normal morphology of the mitochondria when stimulated by external factors, which include nutrient deprivation. However, how the fusion sites are determined remain undefined. Mitochondrial fusion and fission sites have been shown to be induced by a particular lipid environment in the MAMs (77). High calcium concentration also favors the initiation of fission and fusion. Since the formation of MAMs mediates the calcium transfer, the intercellular calcium level regulates Fis1 level and mitochondrial fission (78). For instance, defective mitochondria-ER contacts lead to elevated cytosolic Ca^2+^ levels, thus inducing indirect activation through activating calcineurin phosphatase, which finally leads to mitochondrial fragmentation (72). Future investigations that focus on the physical distance between the ER and the mitochondria will be helpful to better understand the relationship and effects of the alterations in the mitochondrial morphology and ER-mitochondria contact.

### Mitochondrial motility

To meet the energy and calcium demand, mitochondria are transferred along the microtubules. The mitochondria transportation is correlated with mitochondrial Rho GTPase 1 (MIRO1) and MIRO2 (79, 80). Since there is relatively low calcium affinity of MIRO1/2, the motility of mitochondria requires a high calcium content, making MAMs ideal for mitochondria motility (81). In contrast, excess MAMs and calcium may lead to defective axonal mitochondrial transport. The microtubule-dependent mitochondrial transport is mediated by microproteins, TRAK adaptors and dynein/kinesin motors (82). In addition, the delivery of mitochondrial DNA (mtDNA) nucleoids is promoted by the motor protein KIF5B and MAMs *via* mitochondrial dynamic tubulation (83).

## MAMs and autophagy

MAMs have also been associated with the initiation and execution of autophagy, with many autophagy-related genes (ATGs), such as such as ATG14 (autophagosome marker), ATG2/5 (autophagosome-formation marker), Beclin1, and VPS15/34, expressed and localized on the MAMs. TOM40/70 recruits ATG2A to MAMs for phagophore expansion (84). ATG2A stimulates ATG9-vesicle delivery for phagosome expansion and autophagic flux (85). The defects in ER-mitochondria which result from PACS2 and MFN2 knockdown can affect the formation of ATG14 puncta. Besides, the autophagosome formation can be influenced by other MAMs-localizing proteins, such as promyelocytic leukemia protein (PML), by modulating the AMPK/mTOR/ULK1 pathway (86). Mitophagy is responsible for effective removal of damaged and dysfunctional mitochondria, which also occurs at the MAMs (87). As previously reported, mitophagy can mainly be classified into two major pathways: the Parkin-dependent pathways (Figure 2) and adaptor-dependent mitophagy. The adaptor-dependent mitophagy is directly performed by Bcl2 interacting protein 3 (BNIP3), Bcl2 interacting protein 3 like (BNIP3L/NIX), and FUNDC1, with their light chain 3 (LC3) interacting region (LIR) interacting with LC3 to mediate mitophagy (Figure 2) (88–90).

### PINK/Parkin-mediated mitophagy

PINK1 and Parkin pathway is one of the well-studied mitophagy pathways, which is involved in the progression of Parkinson’s disease (89). PINK is often digested by matrix processing peptidases (MPPs) in the mitochondria, which is further degraded in lysosomes (91). However, the degradation of PINK is inhibited under pathological conditions, which aggregates on the OMM (90). The excessive PINK on the OMM results in ubiquitin phosphorylation, and subsequent recruitment of Parkin. Thereafter, the Parkin on the OMM is phosphorylated by PINK, leading to the activation of Parkin polyubiquitinated proteins, such as VDAC1 and p62/SQSTM1 (92). The interaction between the ubiquitinated substrates and LC3 recruits autophagosomal membranes around the mitochondria. Proper activation of the PINK/Parkin-mediated mitophagy is fundamental for homeostasis of intracellular mitochondria, and improper activation of this pathway induces many cardiac diseases (93). *PINK1*-depleted mice exhibit stress overload which induces HF compared with wild type controls. BECN1 is a member of class III phosphatidylinositol 3-kinase (PtdIns3K) complex, which also resides at the MAMs. It modulates the ER-mitochondria contacts and promotes formation of autophagosome precursors (89). BECN1 is abnormally expressed or modified in cardiac hypertrophy and HF (94). Its dysregulation leads to progression of heart diseases by altering autophagy and apoptosis (86).

### FUN14 domain containing 1-mediated mitophagy

FUN14 domain containing 1 participates in the receptor-mediated mitophagy pathway (95). In response to hypoxia, LIR domain in FUNDC1 binds to the LC3 to induce mitophagy. FUNDC1 mediated mitophagy can be modulated by many stress factors and cellular proteins (96). Normally, FUNDC1 is phosphorylated by Src and casein kinase 2 (CK2), which prevents its interaction with LC3 for subsequent mitophagy (97). During hypoxia, FUNDC1 is dephosphorylated by PGAM5, inducing the formation of autophagosomes (98). FUNDC1-mediated mitophagy has been shown to be directly associated with MAMs through binding to IP3R2 for calcium transfer from the ER to the mitochondria and cytoplasm (99). Inhibition of the FUNDC1 expression reduces intracellular calcium level, which leads to suppression of Fis1 level and mitochondrial dysfunction (100). In addition, the expression of FUNDC1 mediates the interaction between the ER and mitochondria as well as the abundance of the MAMs.

## MAMs are closely associated with homeostasis in cardiac hypertrophy

**FIGURE 3:**
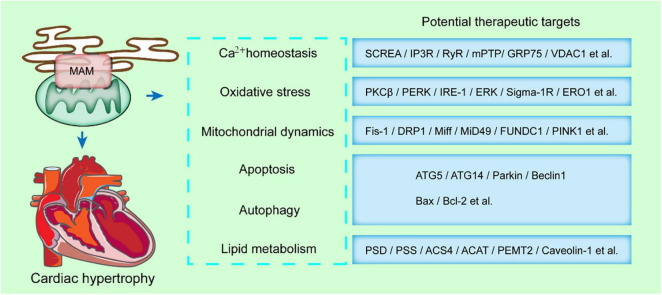
MAMs-enriched proteins present a potential therapeutic strategy in cardiac hypertrophy. MAMs modulates some key cellular processes, such as calcium homeostasis, oxidative stress, mitochondrial dynamics, apoptosis, autophagy, and lipid metabolism. The MAMs-enriched proteins mediating these processes are potential therapeutic targets in cardiac hypertrophy.

When the mitochondrial membrane potential is partially or totally inhibited, calcium homeostasis during contraction is disrupted, and the reduction of shortening degree of cardiomyocytes. Therefore, the calcium transfer from the ER to the mitochondria is closely associated with cardiac contraction. The functions of MAMs in the progression of cardiac diseases have been demonstrated due to its multiple roles in lipid metabolism, calcium transfer, ROS and ER stress, mitochondria dynamics or mitophagy (Figure 3).

Hypertrophic growth of the heart is an adaptive response to hemodynamic stress, which can compensate the cardiac performance and relieve ventricular wall tension as well as oxygen consumption. Physiological hypertrophy results from exercise or pregnancy and is considered a mild and reversible process. However, in aspect to pathological hypertrophy, chronic stressful condition, such as hypertension and valvular disease induce excessive increase in ventricular dimensions, as well as myocardial dysfunction and fibrosis. Worsening of cardiac hypertrophy can result in HF.

During the progression of cardiac hypertrophy or HF, there is alteration of the contact between ER and mitochondria. When stimulated with a hypertrophic factor, norepinephrine, the distance between SR and mitochondria is enhanced, which diminishes the calcium reuptake in cardiac mitochondria. This might be because of the compensatory effect, associated with the increased SR calcium leakage during HF. However, this process might lead to reduced mitochondrial oxidative activity, cardiac metabolic reprogramming to glycolysis, which further aggravate cardiac hypertrophy. Moreover, the deficiency in SR-mitochondria communication and calcium transfer may act as a premise for pathological cardiac hypertrophy in aged mice. Cardiac-specific deletion of RYR2 blocks SR-mitochondria calcium exchange, and results in spontaneous myocardial hypertrophy as well as fibrous hyperplasia in mice. Thus, improper calcium exchange is a typical characteristic in HF. However, there is no conclusive data on the calcium oscillation range. Some previous studies have observed dramatic perturbation of cytosolic cation in HF, which include an increase in Na^+^ levels.

Cardiac hypertrophy and HF can lead to a variety of disorders, such as mitochondrial dynamics. The mitochondrial dynamics in myocardial tissue act as a key aspect in cardiac function. *In vivo* and *in vitro* studies showed MFN2 downregulation such as cardiac hypertrophy which is induced by pressure overload. In addition, OPA1 deficiency is accompanied with mitochondrial fragmentation in both rat and human HF models. Besides, MFN1/2 suppression which was accompanied with mitochondrial network alterations was observed in guinea pig HF models. Norepinephrine can stimulate cardiac hypertrophy and mitochondrial fission by modulating DRP1 (Figure 3). In addition, DRP1 acetylation can activate itself and promote mitochondrial translocation, leading to cardiac hypertrophy and dysfunction induced by excessive lipid supply. In addition, overexpression of defective DRP1 in neonatal rat cardiomyocytes confers a protective effect from noradrenaline-induced mitochondrial network damage and cardiac hypertrophy (Figure 3).

Notably, in the absence of other external stimuli, deficiency in MFN2 is sufficient in inducing hypertrophy in cardiomyocytes. Cardiac-specific MFN2 depletion is associated with moderate myocardial hypertrophy and mild functional deterioration in mice. Several key MAMs-associated proteins have been shown to affect the progression of heart-related diseases. Cardiac-specific MFN2 depletion led to cardiac hypertrophy and moderate diastolic dysfunction (95). Besides, the mice developed obvious systolic dysfunction in response to β-adrenergic stress. In addition, the MFN2 depleted mice exhibited abnormally large and elongated mitochondria and diminished SR-mitochondria contact. MFN2 deficiency in cardiomyocytes also contributed to irregular mitochondrial spatial distribution, low mitochondrial membrane potential and inadequate calcium uptake (96). Activation of Sig-1R by fluvoxamine could protect from abdominal aortic banding or TAC-induced cardiac hypertrophy. FUNDC1 knockout disrupts MAMs and mitochondrial dynamic, eventually compromising cardiac function. Moreover, shortage of OPA1 sensitizes myocardium to mechanical stress. Compared with the wild-type mice, severe cardiac hypertrophy associated with ventricular abnormalities was observed in OPA1^±^ mice, when challenged with transverse aortic contraction. Deletion of YME1L1, an ATP-dependent zinc metalloproteinase, in cardiomyocytes impaired mitochondrial morphology, and led to progressive dilated cardiomyopathy by inducing OPA1 degradation. Therefore, the mitochondrial dynamics is critical in the maintenance of cardiac functions.

Therapy that targets mitochondrial dynamics might act as a promising approach to protect from cardiac hypertrophy and HF. For instance, in a transverse aortic contraction challenged pressure overload mouse model, mdivi-1 administration can ameliorate cardiac fibrosis and left ventricular dysfunction. The inhibitor of mitochondrial division/mitophagy (Mdivi) ameliorates pressure-overload induced HF. PINK1-depleted mice exhibit stress overload which induces HF compared with wild type controls. However, a previous study showed that DRP-1 dependent mitophagy might protect HF induced by pressure overload, thus causing adverse outcome to late-stage HF. The proteins located on the mitochondria also affected the progression of cardiac hypertrophy. Pharmacologic or genetic inhibition of monoamine oxidases, an OMM flavoenzymes, prevents cardiac oxidative stress and contractile dysfunction in mice under pressure-overload stress.

Recent data has emphasized the role of ER stress, such as PERK, in the progression of cardiac hypertrophy and HF (Figure 3). NOGO B, a member of the ER reticulon family, is responsible in defining the morphology of the ER tubular. Also, NOGO B negatively modulates the ER-mitochondria contacts. Suppression of NOGO B contributes to cardiac hypertrophy and cardiac fibroblast activation *via* activation of the PERK/ATF4 signaling pathway and ATF6-mediated ER stress pathway.

## Outlook

Pathological cardiac hypertrophy is one of the main causes of HF, with a highly complex pathogenesis. At present, β Adrenergic receptor antagonists and calcium channel blockers are the main drugs used in the clinical treatment of myocardial hypertrophy. These drugs are often used to alleviate symptoms, reduce myocardial contractility by reducing the acquisition of intracellular calcium ions, prevent arrhythmia and improve energy deficiency. However, the use of these drugs has not been supported by sufficient clinical data, and do not improve natural development of pathological changes in myocardial hypertrophy. Many studies have shown that the abnormal functions of the mitochondria and ER, the energy factory and protein synthesis machinery in cells, respectively, is an important inducement in promoting pathological myocardial hypertrophy. With the rapid development of cell imaging and other technologies, the role of the interaction between different organelles in diseases, especially myocardial hypertrophy, has been demonstrated. The MAMs are involved in maintaining the normal functioning of the ER and mitochondria, and are closely related to cellular lipid metabolism, calcium homeostasis, mitochondrial dynamics, autophagy and apoptosis, ER stress as well as inflammation. As a bridge between the structure and function of the mitochondria and ER, many studies have confirmed MAMs to be potential therapeutic targets for myocardial hypertrophy.

The signal transduction and functional coordination between ER and mitochondria affect the whole life process, which is the most rapidly developing field in biology. Cardiac hypertrophy is closely related to calcium overload and ER stress (101). A variety of drugs have pharmacological effects on myocardial protection by inhibiting ER stress and MAMs to reduce Ca^2+^ release, calcium overload, mitochondrial damage and apoptosis.

Mitochondrial energy metabolism is one of the many mechanisms of cardiac hypertrophy (102). Dysfunctional respiratory chain not only causes allosteric changes in NAD^+^/NADH, resulting in decreased ATP production and REDOX imbalance, but also increases intracellular Ca^2+^ level and ROS generation. And this process is also closely related to the ER. MAMs is undoubtedly a key bridge to link related organelles to regulate ROS and the progression of myocardial hypertrophy.

Interestingly, MAMs mediated biological functions highly overlap with the pathophysiological mechanisms in many diseases. It has been shown that by interfering with the transport of Ca^2+^ by the MAMs anchor protein complex VDAC/IP3R/GRP75, Ca^2+^ overload is inhibited, thereby blocking the opening of mPTP and the release of cytochrome C in the mitochondria. A variety of MAMs related proteins participate in the composition of mPTP and may directly regulate its opening and closing. mPTP opening is an important marker for myocardial cell functions. On the other hand, a variety of drugs or compounds with clear therapeutic effects on cardiovascular diseases have been demonstrated to function through MAMs related proteins. DRP1 acetylation increases its activity and mitochondrial translocation, resulting in cardiomyocyte hypertrophy and dysfunction in response to excessive supply of lipids. Overexpression of inactive DRP1 (DRP1K38A) in cultured neonatal rat cardiomyocytes prevents mitochondrial network damage and noradrenaline-induced hypertrophy. Mdivi-1 treatment can reduce cardiac fibrosis and left ventricular dysfunction in a mouse model of pressure overload induced by transverse aortic contraction. In addition, highly specific agonists of Sig-1R have been shown to modulate cardiomyocytes’ contractility. In addition, Sig-1R activation represses hypertrophy and cardiomyocyte injury induced by angiotensin II (Figure 3). Berberine II can reduce the permeability of mPTP and inhibit apoptosis by downregulating the expression of VDAC1 and alleviate cerebral ischemia/reperfusion injury. Besides, sinomenine inhibits the activation of NLRP3 inflammatory bodies and plays a neuroprotective role in cerebrovascular disease. On the other hand, polyunsaturated fatty acids can reduce neonatal hypoxic-ischemic brain injury by affecting MAMs protein PSS1. In conclusion, our analysis demonstrated that MAMs can participate in the multifaceted pathological process of heart related diseases, especially myocardial hypertrophy, which provides evidence for MAMs as a potential target in the treatment of myocardial hypertrophy.

## Author contributions

YL, YJ, and YY conceptualized and wrote the manuscript and created figures. YL, YY, and HL contributed to the writing of the manuscript. YL, HL, PZ, and YY reviewed and modified the manuscript. All authors approved the final version of the manuscript.
